# The protective role of GPX4 in naïve ESCs is highlighted by induced ferroptosis resistance through GPX4 expression

**DOI:** 10.1016/j.redox.2025.103539

**Published:** 2025-02-10

**Authors:** Seokwoo Park, Mihn Jeong Park, Eun-Ji Kwon, Ji-Young Oh, Yeon-Joon Chu, Han Sun Kim, Sunghyouk Park, Tae Ha Kim, Sung Won Kwon, Yon Su Kim, Hyuk-Jin Cha

**Affiliations:** a Department of Biomedical Sciences, Seoul National University College of Medicine, Seoul, Republic of Korea; b Department of Internal Medicine, Seoul National University College of Medicine, Seoul, Republic of Korea; c Department of Internal Medicine, Seoul National University Bundang Hospital, Seongnam, Republic of Korea; d College of Pharmacy, Seoul National University, Seoul, Republic of Korea; e Natural Products Research Institute, College of Pharmacy, Seoul National University, Seoul, Republic of Korea; f Research Institute of Pharmaceutical Sciences, Seoul National University, Seoul, Republic of Korea

**Keywords:** Ferroptosis, Naïve pluripotency, Primed pluripotency, Glutathione peroxidase 4 (GPX4)

## Abstract

Ferroptosis, a form of oxidative cell death mediated by lipid peroxidation, is strictly regulated by glutathione peroxidase 4 (GPX4). Knockout of *Gpx4* results in embryonic lethality, highlighting its essential role in development. *In vitro*, mouse embryonic stem cells (mESCs), which represent the naïve pluripotent state, require β-mercaptoethanol (bME) to prevent cell death, unlike human embryonic stem cells, which represent the primed state. We hypothesized that naïve pluripotency is linked to a heightened susceptibility to ferroptosis due to unique metabolic demands and redox imbalances. In this study, we found that bME deprivation induces ferroptosis in naïve ESCs, as evidenced by lipid peroxidation; ferroptosis, however, is less evident in primed ESCs. Mechanistic analyses revealed that active oxidative phosphorylation (OXPHOS) in naïve ESCs increased mitochondrial reactive oxygen species. Consistent with the upregulation of *Gpx4* transcripts and OXPHOS-associated gene sets seen in the inner cell mass of blastocysts, stable GPX4 expression conferred resistance to ferroptosis induced by bME withdrawal. These results suggest that the unique redox and metabolic landscape of naïve ESCs highlits a potential requirement for GPX4 in maintaining naïve pluripotency, providing insights into early developmental processes and vulnerabilities.

## Highlights

• Ferroptosis vulnerability of naïve ESCs necessitates bME supplementation.
• A distinct cellular context in naïve ESCs creates this vulnerability.
• GPX4 activity is required during embryo development to prevent ferroptosis.

## 1 Introduction

Human embryonic stem cells (hESCs) have received greater research focus than mouse ESCs (mESCs) due to their potential clinical applications [1]. Notably, the cellular and molecular dissimilarities of these cells in terms of morphology, epigenetics, glucose metabolism, and signaling pathways are increasingly recognized as resulting from their different pluripotency states—naïve-like for mESCs and primed-like for hESCs—rather than any species-specific distinction [2,3]. The distinct naïve and primed pluripotent states recapitulate the cellular and molecular characteristics of pre- and post-implantation embryos, respectively [[2], [3], [4]]. Thus, isogenic pairs of mouse/human ESCs in both naïve and primed states have been employed to elucidate the drastic biological transitions that occur during early embryonic development [[5], [6], [7]]. In particular, while naïve ESCs utilize both glycolysis and mitochondrial oxidative phosphorylation (OXPHOS), the transition to primed state accompanies a metabolic switch to glycolysis-dependent glucose usage [8]. This resembles the drastic shift observed during early embryo development [9]. In addition, lipid droplet prevalence and the high dependency on fatty acid synthesis and its oxidation in the oocyte to blastocyst stages underscore the role of fatty acids as an integral energy source before implantation [10]. Recent studies investigating the necessity of fatty acid synthesis and oxidation for ESC survival have further highlighted the unique metabolic adaptations employed to meet the high energy demands of embryonic development [11,12]. In addition, reactive oxygen species (ROS) are formed as a byproduct of mitochondrial OXPHOS [13], which necessitates the presence of diverse oxygen-scavenging antioxidants in oviductal fluids, such as vitamins A, C and E, taurine, glutathione (GSH) and cysteamine [14]. Accordingly, maintaining mESCs in culture requires a constant antioxidant supply (e.g., of β-mercaptoethanol [bME]), as lack of antioxidants induces massive p53-dependent cell death [15]. Contrastingly, antioxidant supplementation is unnecessary for hESC culture [16]. The reason for this differential antioxidant demand yet remains unclear.

Ferroptosis, a recently identified form of nonapoptotic cell death, occurs through iron-dependent lipid peroxidation accumulation [17]. The pathological relevance of this process has been actively examined in degenerative diseases and cancers [18]. Vital defense systems include intracellular GSH, synthesized from imported cysteine (through System X_C_-: XCT, a cystine/glutamate antiporter encoded by *SLC7A11* and glutathione peroxidase 4 (GPX4) which uses GSH as a substrate to reduce toxic lipid peroxides (L-OOH) into non-toxic lipid alcohols (L-OH) [19]. Thus, XCT and GPX4 present promising molecular targets for ferroptosis inducers, aimed at disabling the intrinsic ferroptosis defense system to develop novel anti-cancer drugs [20]. Besides GSH and GPX4, factors such as redox imbalance, iron metabolism, p53 activity, signaling pathways, polyunsaturated fatty acid levels, and a variety of other cellular components have been shown to influence ferroptosis vulnerability in cell line models [21].

This study demonstrated that ferroptic cell death induced by deprivation of bME, specifically occurring in naïve ESCs but not primed ESCs, was completely inhibited by either ferrostatin treatment or GPX4 stable expression. Distinct metabolic and cellular characteristics in naïve ESCs, mitochondrial ROS, and high expression of *Tfr1* (a transferrin receptor) together prompt high ferroptosis susceptibility and necessitate a constant antioxidant supply. These findings mirror the critical role of GPX4 during normal early embryonic development, as evidenced by the embryonic lethality observed in *Gpx4* knockout embryos.

## 2 Materials and methods

### 2.1 Cell culture

Naïve and primed mESCs were maintained as described previously [22]. Briefly, naïve mESCs were cultured on 0.5 % gelatin-coated culture dishes. DMEM high glucose (Gibco) media was used supplemented with 15 % FBS (Gibco), 1 % MEM-nonessential amino acids (Gibco), 1 % Glutamax (Gibco), 0.1 mM β-mercaptoethanol (bME) (Gibco), 1000 U/ml mouse leukemia inhibitor factor (Millipore, Merck), 1 μM MEK1 inhibitor PD0325901 (MedChem Express, Monmouth Junction, NJ), and 3 μM GSK3β inhibitor CHIR99021 (MedChem Express). Primed mESCs were maintained on Matrigel (Corning#354277)-coated dish. Basal media was DMEM/F12 (Gibco) which was supplemented with 15 % KOSR (Gibco), 1 % MEM-nonessential amino acids (Gibco), 1 % Glutamax (Gibco), 10 ng/ml murine bFGF (Peprotech), 20 ng/ml murine Activin-A (Peprotech). Cells were cultured at 37 °C supplied with 5 % CO_2_ and added with 0.1 % Gentamycin (Gibco). Isogenic pairs of J1-PJ1 and OG2-POG2 mESCs representing naïve and primed pluripotent states were used in the study. To exclude the potential bias arising from environmental lipid content on differential metabolism, naïve mESCs were cultured in a medium, where 15 % FBS was substituted with 15 % KOSR, mirroring the culture condition of primed mESCs. Experiments were conducted on naïve cells that had adapted to the KOSR-supplemented medium for a minimum of three passages. J1 line was purchased from the American Tissue Culture Collection (ATCC, Manassas, VA). OG2 transgenic mESC line was kindly provided by Dr. Hans R. Schöler.

### 2.2 Establishment of naïve ESCs with stable expression of GPX4

A PiggyBac-based expression vector (VB900123-7333ggr: pPB[Exp]-Puro-CMV > mGpx4[NM_001037741.4]:IRES:TagBFP2) was purchased from Vector Builder and used to generate a stable cell line as described previoulsy [23]. Cells were co-transfected with the GPX4-expressing vector and a PiggyBac transposase plasmid using Lipofectamine 3000 (Thermo Fisher Scientific) according to the manufacturer's protocol. After 48 h, cells were selected with puromycin (1 μg/ml) to enrich for transgene-positive cells. Stable integration and overexpression of GPX4 were confirmed by qPCR and Western blot analysis.

### 2.3 Cell death assay

To induce ferroptosis, 0.1 mM β-mercaptoethanol (bME) was removed from the standard culture media of naïve mESCs for 48 h. For time-lapse images, the specific timelines were indicated in the figures. Cell death via ferroptosis was quantified by flow cytometric analyses of 7-AAD-positive cells. After treatment with the indicated reagents, cells were harvested, washed twice with PBS, and stained with 7-AAD (BD Bioscience, #559925) for 45–60 min at room temperature in the dark. Flow cytometric analysis was performed using a FACS Calibur instrument (BD Bioscience). The proportion of live cells (7-AAD-negative) was calculated to assess cell viability. To explore the specific types of cell death involved after the bME deprivation, inhibitors such as ferrostatin-1 (Fer-1, 1 μM) and zVAD (20 μM) were treated for 24 h before harvest to block ferroptosis and apoptosis, respectively. For apoptosis control experiments, cells were treated with etoposide (Eto, 1 μM). To assess cell viability in etoposide-treated naïve mESCs, 7-AAD and Annexin V dual-staining was performed as per the manufacturer's protocol (BD Biosciences, Annexin V-FITC Apoptosis Detection Kit, #556547). Briefly, cells were harvested, washed twice with PBS, and resuspended in 1 × binding buffer at a concentration of 1 × 10^6 cells/mL. Subsequently, 5 μL of Annexin V-FITC and 5 μL of 7-AAD were added to 100 μL of cell suspension. The cells were incubated for 15 min at room temperature in the dark. After staining, 400 μL of 1 × binding buffer was added, and samples were immediately analyzed by flow cytometry using a FACS Calibur instrument (BD Bioscience). For all flow cytometry analyses, doublets were excluded based on forward scatter (FSC) and side scatter (SSC) gating. A minimum of 10,000 target cells were recorded for each independent experiment.

### 2.4 Flow cytometric analyses of lipid peroxidation

To investigate the extent of membrane lipid peroxidation, cells were deprived of bME for 24–48 h. Inhibitors targeting specific cell death pathways including ferrostatin-1 (Fer-1, 1 μM) and zVAD (20 μM) were applied for 24 h prior to analyses. Cells were dissociated with Accutase (#561527, BD Bio-sciences) and washed twice with DPBS. Cells were counted and incubated with BODIPY 581/591C11 (5 μM, Invitrogen #D3861) in DPBS at 37 °C for 30 min. Following incubation, cells were washed with DPBS and subjected to flow cytometric analyses using a FACS Calibur instrument (BD Bioscience). For the detection of lipid peroxidation in mitochondrial membrane, cells were stained with MitoPerOx (5 μM; Cayman Chemical, #18798) using the same procedure.

### 2.5 MitoSox red staining

To evaluate mitochondrial superoxide levels, naïve and primed mESCs cultured under the standard conditions were harvested. The cell suspensions were incubated with MitoSox Red (5 μM, Thermo Fisher Scientific, #M36008) in DPBS at 37 °C for 10 min in the dark. as per the manufacturer's protocol. After staining, samples were washed with DPBS and analyzed immediately using a FACS Calibur instrument (BD Biosciences).

### 2.6 Live cell imaging

Live cell images were captured by JuLI™ Stage (NanoEnTek Inc.). The acquired images were further processed and analyzed with JuLI^TM^STAT software according to the manufacturer's instructions.

### 2.7 Measurement of the reduced to oxidized glutathione

The GSH/GSSG ratio was determined using the GSH/GSSG-Glo Assay (Promega, WI, USA). Cells were treated with 50 μl of Total Glutathione Lysis Reagent or Oxidized Glutathione Lysis Reagent per well and agitated at room temperature for 5 min. Subsequently, 50 μl of Luciferin Generation Reagent was added to each well, and the cells were incubated at room temperature for 30 min. Following that, 100 μl of Luciferin Detection Reagent was added to each well. Luminescence was measured using a microplate reader (SpectraMax® i3x Multi-Mode) to determine the GSH/GSSG ratio.

### 2.8 Luciferase assay

Cells were co-transfected with Srebp reporter vector and pRL vector using Lipofectamine 2000 reagent (#11668019, Invitrogen). After 3 h of incubation with 1 × passive lysis buffer followed by centrifugation, supernatant from the cell lysate was acquired. The supernatant was used for the reaction with LARII and Stop & Glo reagent. Luciferase assay was performed using Dual Luciferase Reporter Assay System Kit (#E1980, Promega), and detected by SpectraMax® i3x Multi-Mode Microplate Reader.

### 2.9 CoQ analysis by LC-MS/MS

Naive and Primed samples (n = 7) were analyzed by LC-MS/MS. An Agilent 6460 triple-quadrupole mass spectrometer was coupled to an Agilent 1290 UPLC as the analysis instrument. LC column was Acquity UPLC BEHC18, 1.7 μm × 2.1 mm x 50 mm (Waters, Massachusetts, USA) and maintained at 40 °C. Mobile phase A was ACN: water (60:40, v/v) with 0.1 % formic acid, while mobile phase B was ACN: IPA (10:90, v/v) with 0.1 % formic acid. The flow rate was 0.3 mL/min. The mobile phase gradient was as follows: B was 15 % at 0 min, 30 % at 4 min, 48 % at 5min, 82 % at 22 min, 99 % from 23 min to 24 min, and 15 % from 24.2 min to 30 min. Total run time was 34 min including 4 min post-run. The injection volume was 5 μL. The gas temperature was 325 °C. Sheath gas temperature was 350 °C at 11 L/min. The nebulizer gas was 35 psig. The capillary voltage was 3500 V. MRM mode was used as a mass spectrometer. The ESI ionization mode was positive. The transition was as follows. Coenzyme Q10: *m*/*z* 863.7 → 197.2, fragmentor = 240 V, CE = 42 V, dwell = 200 ms.

### 2.10 Generation and preprocessing of RNA sequencing data

Total RNA was isolated from J1, PJ1, OG2 and POG2 cells in duplicates using Trizol according to the manufacturer's instructions. For library construction, we used the TruSeq Stranded mRNA Library Prep Kit (Illumina, San Diego, CA). Briefly, the strand-specific protocol included the following steps [1]: strand cDNA synthesis [2], strand synthesis using dUTPs instead of dTTPs [3], end repair, A-tailing, and adaptor ligation, and [4] PCR amplification. Each library was then diluted to 8 pM for 76 cycles of paired-read sequencing (2 × 75 bp) on an Illumina NextSeq 500 following the manufacturer's recommended protocol. Read quality was assessed using FastQC (v) and poor-quality bases (Phred score <20) were eliminated using TrimGalore (v0.6.6). Trimmed reads were aligned to the human reference genome (GRCm39) using the STAR aligner (v2.7.9a) with default parameters. Gene-level expression values such as transcripts per million (TPM) and read counts were calculated using RSEM (v1.3.3.) with human annotation (GRCm39.104). FASTQ format files, gene-level count data, and TPM of all samples are available in supplement data. Among the 24482 genes, 19585 protein-coding genes were utilized for subsequent analysis. Differential gene expression analysis was performed using the ‘DESeq2’ package (v3.15) in R (v4.2.1). Transcripts were considered significant if their fold change was above or below 1 (in log2 scale) and the p-value corrected by FDR was below 0.05. For GSEA, differential gene expression analysis between groups (Naïve vs Primed) was performed using the R package ‘DESeq2’, yielding a ranked list of genes. Using the gene list as the input, GSEA was run via the R package ‘fgsea’ for public annotated gene sets of interest (e.g., KEGG, Hallmarks, Wikipathways and Reactomes pathways, oncogenic signatures). We utilized the differential expressed genes to obtain KEGG mapping data related to “Ferroptosis”. Genes exhibiting high expression in the naïve condition were visually represented using the color red, while genes with high expression in the primed condition were depicted in green.

### 2.11 Re-analysis of published transcriptomics data

The current publication utilized processed data from the following publications: Siqin Baoet al. (GSE99491), Masaki Kinoshita et al. (GSE131555), and Alex Neagu et al. (GSE105762). Data was downloaded directly from GEO repository. Differential gene expression analysis was performed using the ‘DESeq2’ package (v3.15) in R (v4.2.1). Transcripts were considered significant if their fold change was above or below 1 (in log2 scale) and the p-value corrected by FDR was below 0.05. The genes used for PCA were selected based on their identification as differentially expressed genes (DEGs) in all transcriptome analyses conducted.

### 2.12 Alexa Fluor 568 transferrin staining and quantification

For Alexa Fluor® 568 staining of *in vitro* cultured cells, cells were seeded in a Confocal dish(SPL, cat# 211350). After two days of culture, cells were kept on ice for 10 min before staining. Cells were washed with cold DPBS containing 20 mM Glucose and 1 % BSA. Cells were incubated at 37 °C for 20min with 25 μg/mL of Alexa Fluor® 568 (life technologies, Cat# T23365) in DPBS containing 20 mM Glucose and 1 % BSA. After washing cells in DPBS containing 20 mM Glucose and 1 % BSA, fluorescence images of live cells were captured by Confocal Scope TCS8(LEICA). Fluorescence images were analyzed by Fiji software.

### 2.13 Teratoma formation assay

To evaluate the pluripotency of the generated mESC lines, wild-type and GPX4-overexpressing naïve mESCs were subcutaneously injected into immunodeficient Balb/c-nu mice to induce teratoma formation. After a defined period, the teratomas were excised, fixed, and subjected to histological analysis. Pluripotency was assessed by confirming the presence of derivatives from all three germ layers through H&E staining.

### 2.14 Quantification and statistical analysis

The quantitative data are expressed as the mean values ± standard deviation (SD). Statistical tests were performed using the PRISM software, and specific methods were described in the Figure legends. P values less than 0.05 were considered statistically significant, p < 0.05(∗), p < 0.01(∗∗), p < 0.001(∗∗∗), and p < 0.0001(∗∗∗∗).

## 3 Results

### 3.1 Cell death of naïve but not primed ESCs triggered by bME depletion

**Fig. 1:**
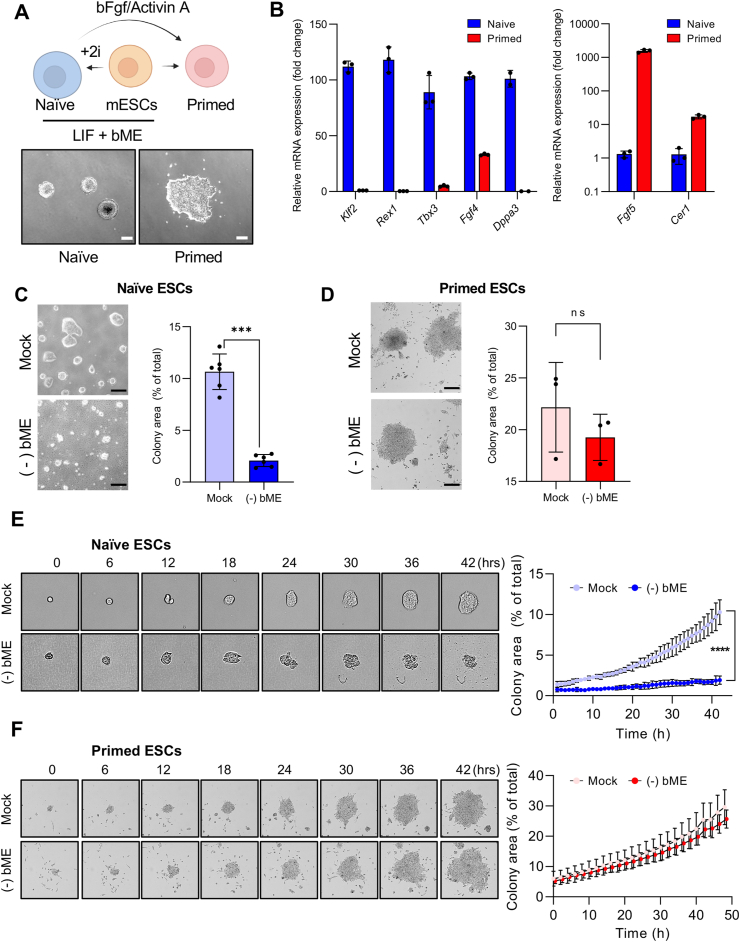
**Cell death of naïve but not primed ESCs triggered by bME depletion (A)** Graphical illustration of the culture conditions for obtaining naïve and primed ESCs from mouse ESCs. Representative brightfield images show the dome-shaped colony characteristic of naïve ESCs and the flat epithelial morphology of primed ESCs. LIF, leukemia inhibitory factor. Scale bars, 200 μm. **(B)** Quantification of mRNA expression for naïve- (left) and primed-specific (right) markers in naïve and primed ESCs. Biological replicates: n = 2 for *Dppa3* and n = 3 for all others. **(C)** Microscopic images (left) and quantification of colony area (right) of naïve ESCs cultured in the presence (Mock) or absence of bME [(−) bME] for 48 h. Colony area was calculated as a percentage of the total culture surface area. Scale bars, 200 μm. Biological replicates: n = 6 per condition. **(D)** Microscopic images (left) and quantification of colony area (right) of primed ESCs cultured in the presence (Mock) or absence of bME [(−) bME] for 48 h. Colony area was calculated as a percentage of the total culture surface area. Scale bars, 200 μm. Biological replicates: n = 3 per condition. **(E**–**F)** Time-lapse images (left) and quantification (right) of colony area (%) of naïve ESCs **(E)** and primed ESCs **(F)** cultured in the presence (Mock) or absence of bME [(−) bME]. Representative images show colony morphology at the indicated time points. Colony area was calculated as a percentage of the total culture surface area. Biological replicates: n = 6 for naïve and n = 3 for primed ESCs. Data show mean ± s.d. **(B–F)**. P values were calculated using a two-tailed *t-*test **(C, D)** or two-way ANOVA **(E, F)**. ∗∗∗p < 0.001; ns, not significant.

**Fig.S1:**
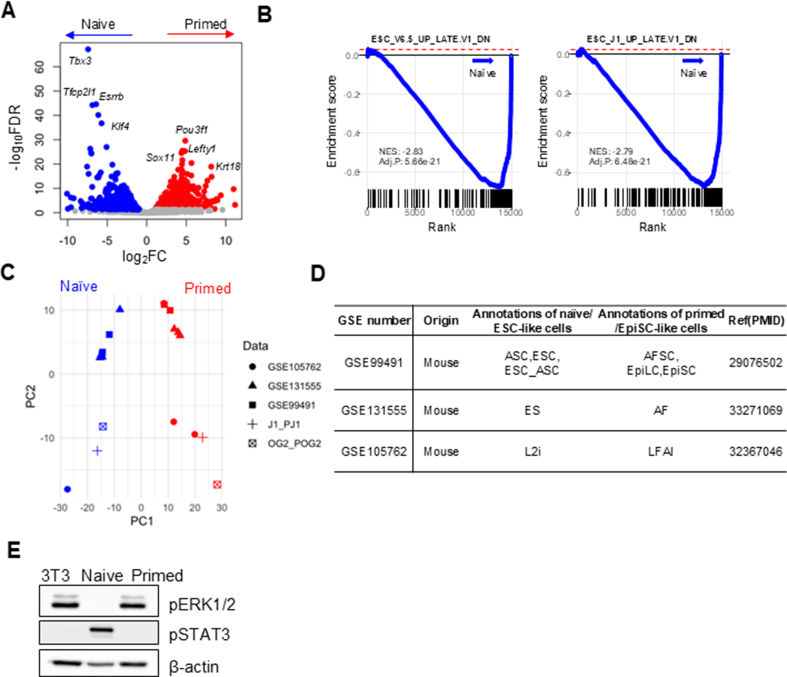


Naïve ESCs are readily achieved by adding two chemical inhibitors (MEK1 inhibitor: iMEK1 and GSK3β inhibitor: iGSK3β) upon exposure of leukemia inhibitor factors (LIF; hereafter LIF/2i) with bME [5]. Conversely, primed ESCs require prolonged culture with basic fibroblast growth factors (bFGF) and activin A (Fig. 1A, top). Naïve and primed ESCs are distinguishable by both cellular and molecular features, respectively exhibiting dome-shaped colonial and flat disc morphologies along with typical mRNA expression of marker genes (Fig. 1A bottom and 1B). Furthermore, transcriptome and gene set enrichment analyses from isogenic pairs of naïve and primed ESCs validated their expression of pluripotency genes (Figs. S1A and S1B). In addition, previously reported transcriptomes of naïve and primed pairs (GSE99491, GSE131555, and GSE105762; Fig. S1D) were used to accurately classify the transcriptomes from two independent naïve (J1 and OG2 mESCs) and primed (PJ1 and POG2 mESCs) pairs (Fig. S1C). Distinct signals corroborated specific naïve and primed pluripotent states of the isogenic pairs (phosphorylated STAT3 level represents JAK/STAT3 signaling upon LIF in naïve ESCs; phosphorylated ERK1/2 level represents MEK1-ERK1/2 signaling upon bFGF in primed ESCs) (Fig. S1E). On bME deprivation, membrane rupture accompanied by necrotic cell death, was observed in naïve ESCs, whereas such membrane damage was not observed in primed ESCs (Fig. 1C and D; Movies S1A and S1B). Consistently, growth inhibition resulting from the absence of bME was manifested in naïve ESCs but not in primed ESCs (Fig. 1E and F).

### 3.2 Cell death of naïve ESCs upon bME depletion occurs through ferroptosis

**Fig. 2:**
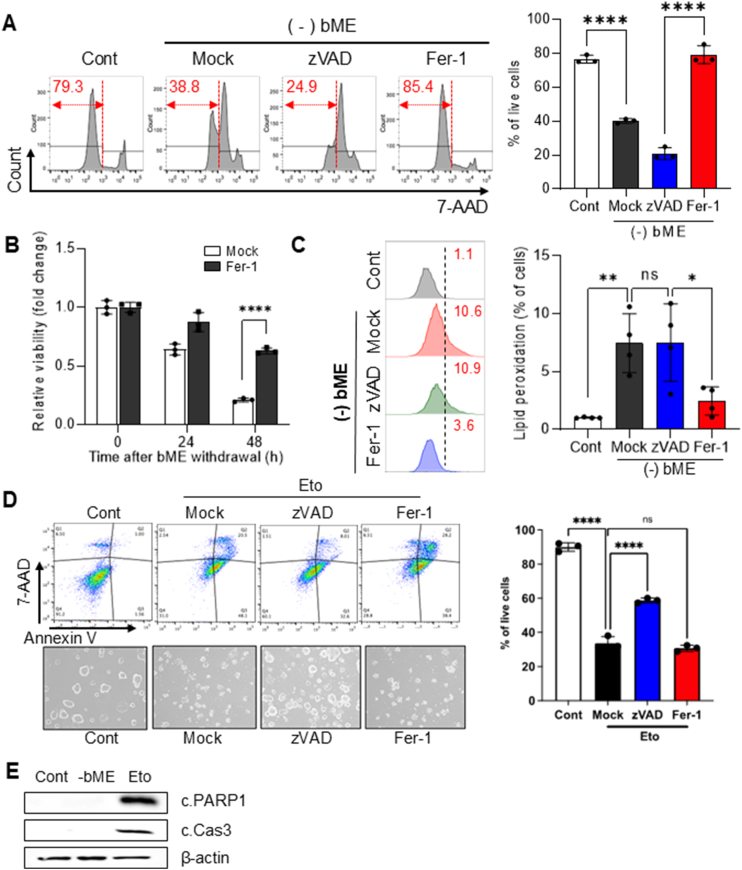
**Cell death of naïve ESCs upon bME depletion occurs through ferroptosis (A)** Flow cytometric analyses of 7-AAD negative live populations in naïve ESCs cultured with (Cont) or without bME [(−) bME], treated with vehicle (Mock), pan-caspase inhibitor (zVAD, 20 μM), or ferrostatin-1 (Fer-1, 1 μM) for 24 h. The percentage of live cells is quantified (right). Biological replicates: n = 3 for each group. **(B)** Time-dependent cell viability of bME-deprived naïve ESCs cultured in the absence (Mock) or presence of Fer-1 (1 μM) for 24 h and 48hrs, assessed using 7-AAD staining. Biological replicates: n = 3 for each group. **(C)** Lipid peroxidation in bME-deprived naïve ESCs assessed by flow cytometry using C11-BODIPY 581/591 staining. Cells were cultured without (Mock) or with bME [(−) bME], and treated with either zVAD (20 μM) or Fer-1 (1 μM) for 24 h. Biological replicates: n = 3 for each group. **(D)** Flow cytometric analysis (top left) and corresponding microscopic images (bottom left) of naïve ESCs treated with the apoptosis inducer etoposide (Eto) in the presence or absence of zVAD (20 μM) or Fer-1 (1 μM) for 24 h. Live cells were assessed by 7-AAD and Annexin V staining. The percentage of live cells is shown (right). Biological replicates: n = 3 for each group. **(E)** Immunoblot analysis of cleaved PARP1 (c.PARP1) and cleaved caspase-3 (c.Cas3) in naïve ESCs cultured with bME (Cont), without bME (-bME), or treated with 1 μM etoposide (Eto, cultured in the presence of bME) for 24 h. β-actin was used as a loading control. Data show mean ± s.d. **(A**–**D)**. *P* values were calculated using one-way ANOVA with Tukey test for post-hoc analyses **(A, C, D)** or two-tailed *t*-test **(B)**.

Previous studies indicated that oxidative stress upon bME withdrawal induces p53-dependent apoptosis [15]. However, unexpectedly, we observed that cell death from bME deprivation was only attenuated by treatment with ferrostatin-1 (Fer-1), a typical ferroptosis inhibitor, rather than with zVAD, a pan-caspase inhibitor (Fig. 2A). The rescue effect of Fer-1 on naïve ESCs 48 h after bME withdrawal implies that this cell death may result from ferroptosis rather than apoptosis (Fig. 2B). To further validate this unexpected finding, we measured the level of lipid peroxidation, a hallmark of ferroptosis, using C11-BODIPY 581/591 in naïve ESCs after bME withdrawal. We found that peroxidized lipids were markedly increased by bME withdrawal, an effect consistently suppressed by Fer-1 but not by zVAD (Fig. 2C). In contrast, apoptosis induced by etoposide (Eto) in naïve ESCs was specifically inhibited by zVAD, a pan-caspase inhibitor, but not by Fer-1, a ferroptosis inhibitor (Fig. 2D). The cell-death-specific effects of these small molecule inhibitors (Fig. 2B for ferroptosis and Fig. 2C for apoptosis) confirm that bME depletion induces ferroptosis rather than apoptosis. Notably, while Eto treatment resulted in distinct cleavage of PARP1 and caspase 3, hallmark indicators of apoptotic cell death, bME depletion-induced cell death was not associated with these apoptotic events (Fig. 2E).

### 3.3 Naïve-specific lipid metabolism: the mevalonate pathway is attenuated in naïve ESCs

**Fig. 3:**
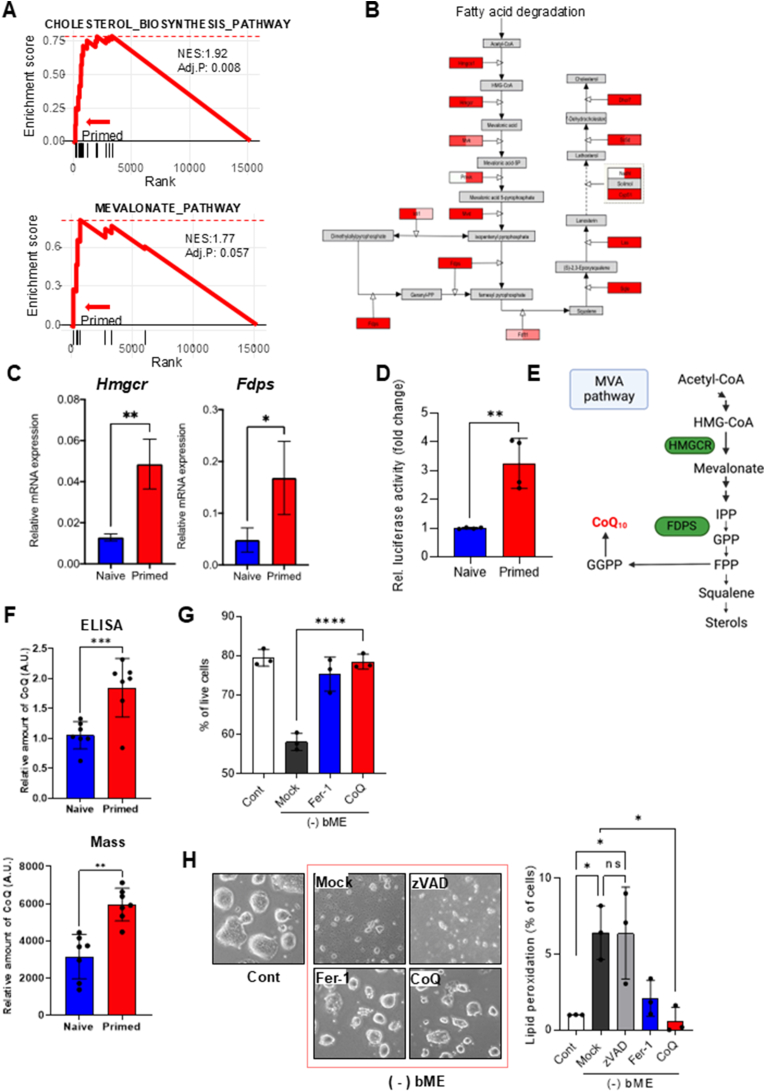
**Naïve-specific lipid metabolism: the mevalonate pathway is attenuated in naïve ESCs (A)** Enrichment plots from GSEA of RNA-seq data illustrating reduced activity of the mevalonate pathway in naïve ESCs compared to primed ESCs. The gene sets were derived from the Wikipathway database. **(B)** Schematic of the mevalonate pathway and downstream processes, highlighting key enzymes in cholesterol and isoprenoid biosynthesis. Red boxes indicate enzymes downregulated in naïve ESCs versus primed ESCs, based on RNA-seq analysis. The red intensity reflects fold change magnitude. The left and right halves of each box show fold changes from the J1/P-J1 and OG2/P-OG2 ESC pairs, respectively. **(C)** Relatively lower mRNA levels of *Hmgcr* and *Fdps* in naïve compared to primed ESCs, determined by qRT-PCR. Biological replicates: n = 3 per condition. **(D)** Relatively lower luciferase activity of sterol regulatory element (SRE) reporters in naïve compared to primed ESCs. Biological replicates: n = 2 for J1/P-J1 pairs and n = 2 for OG2/P-OG2 pairs. Data are normalized to Renilla luciferase and presented as fold change. **(E)** Schematic of the mevalonate (MVA) pathway and its downstream processes involved in isoprenoid and cholesterol biosynthesis. HMGCR and FDPS (green) are key enzymes downregulated in naïve ESCs, as shown by qRT-PCR. CoQ (red) acts as an antioxidant that inhibits ferroptosis. **(F)** Reduced amounts of CoQ in naïve ESCs, determined using ELISA (upper) and mass spectrometry (lower). Biological replicates: n = 7 per condition for each method. **(G)** Viabilities of control and bME-depleted naïve ESCs for 48 h, determined by 7-AAD staining. bME-depleted cells were treated with vehicle, Fer-1 (1 μM), or idebenone (CoQ, 2 μM) for 24 h before harvest. Biological replicates: n = 3 per condition. **(H)** Representative bright-field images (left) and quantification of lipid peroxidation (right) in naïve ESCs cultured with or without bME and treated with zVAD (20 μM), Fer-1 (1 μM), or idebenone (CoQ, 2 μM) for 24 h. Lipid peroxidation levels (% of cells) were assessed using flow cytometry after staining with C11-BODIPY 581/591. Biological replicates: n = 3 per condition. Data show mean ± s.d. **(C, D, F–H)**. *P* values were calculated using two-tailed *t*-test **(C, D, F, G)** or one-way ANOVA followed by Tukey's post-hoc test **(H)**. ∗p < 0.05, ∗∗<0.01, ∗∗∗p < 0.001, ∗∗∗∗p < 0.0001; ns, not significant.

**Fig.S2:**
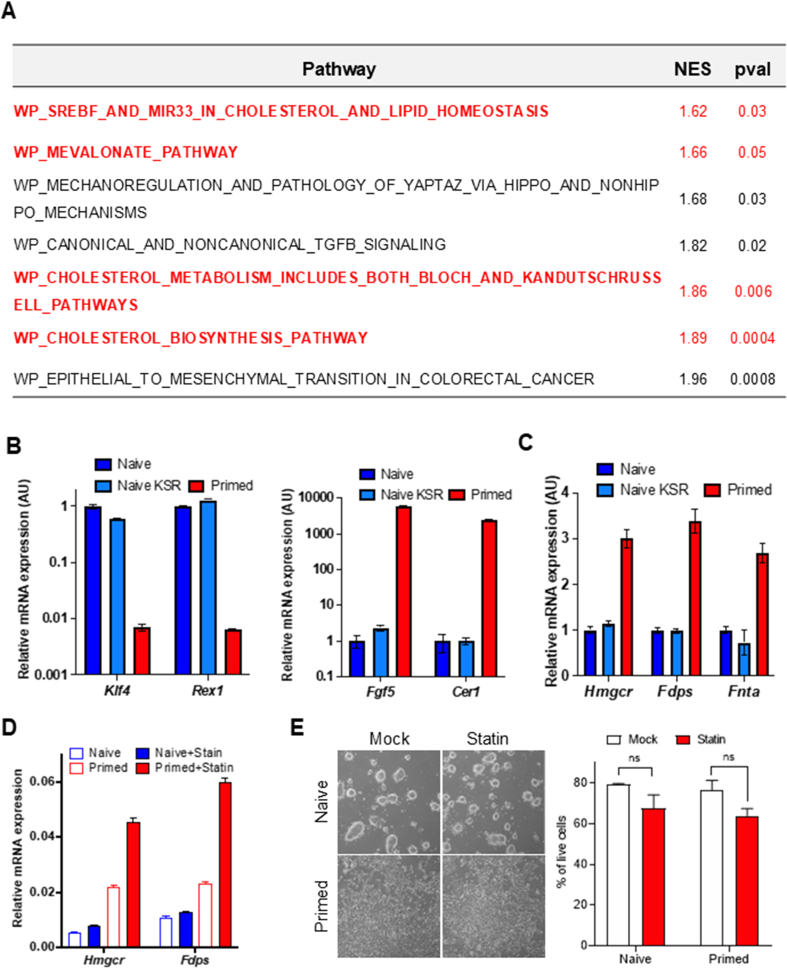


Next, we analyzed RNAseq datasets from naïve and primed ESCs to understand their distinct underlying molecular mechanisms and the naïve-specific ferroptosis sensitivity. A prominent gene set significantly altered between naïve and primed ESCs (Fig. S2A) was associated with ‘Cholesterol Biosynthesis’ or the ‘Mevalonate (MVA) Pathway’ (Fig. 3A and B). This *in silico* prediction was validated through qRT-PCR of two genes encoding essential MVA pathway enzymes, *Hmgcr* and *Fdps*, which are regulated by activity of sterol regulatory element-binding protein 2 (SREBP2) [24] and the sterol-regulatory element, a central motif for lipid biosynthesis; the results supported lower expression in naïve ESCs (Fig. 3C). Consistently, in independent pairs of naïve (J1 and OG2) and primed (PJ1 and POG2) ESCs, transcriptional luciferase activity associated with the sterol-regulatory element was significantly lower in naïve ESCs compared with their primed counterparts (Fig. 3D).

Unlike naïve ESCs, primed ESCs exhibit a high tendency for spontaneous differentiation [25], reflecting their 'primed' state for differentiation [26]. To maintain primed pluripotency, a chemically defined medium such as KnockOut Serum Replacement (KSR) is required [27]. To assess whether exclusive use of KSR influences ESC metabolism, naïve ESCs were cultured for several passages in a KSR-based medium supplemented with LIF/2i (termed naïve KSR). Culture in the naïve KSR condition was found to both preserve naïve-specific pluripotency features (Fig. S2B) and not alter expression of MVA pathway genes, including *Hmgcr*, *Fdps*, and *Fnta* (Fig. S2C).

Given the regulatory roles of the MVA pathway and its downstream products (e.g., ubiquinone/coenzyme Q10 [CoQ] and 7-dehydrocholesterol [7-DHC]) in ferroptosis inhibition [18,[28], [29], [30]], we hypothesized that the antioxidant indispensability (i.e., high demand of bME for survival) observed in naïve ESCs, but not primed ESCs (Fig. 2), could be attributed to attenuated MVA pathway activity (Fig. 3). Supporting this hypothesis, atorvastatin (statin) treatment of naïve ESCs to disrupt the MVA pathway failed to induce specific MVA pathway genes, such as *Hmgcr* and *Fdps*, unlike in primed ESCs (Fig. S2D); this suggests a lack of SREBP2-dependent gene response in naïve ESCs. Consistently, CoQ, a key product of the MVA pathway (Fig. 3E) and critical for ferroptosis defense [28], was diminished in naïve ESCs, as indicated by lower intracellular CoQ levels on ELISA (Fig. 3F, top) and mass spectroscopy (Fig. 3F, bottom). Accordingly, attenuated MVA pathway activity in naïve ESCs may underlie their heightened dependency on exogenous antioxidants for survival. Thereby, the oxidized CoQ analog idebenone was as effective as Fer-1 in preventing both cell death (Fig. 3G) and lipid peroxidation (Fig. 3H) in bME-depleted conditions. These findings indicate that the attenuated MVA pathway activity, which results in insufficient CoQ, disrupts anti-ferroptotic defense mechanisms.

### 3.4 Redox imbalance from oxidative phosphorylation and high TfR1 expression predispose naïve ESCs to ferroptosis

**Fig. 4:**
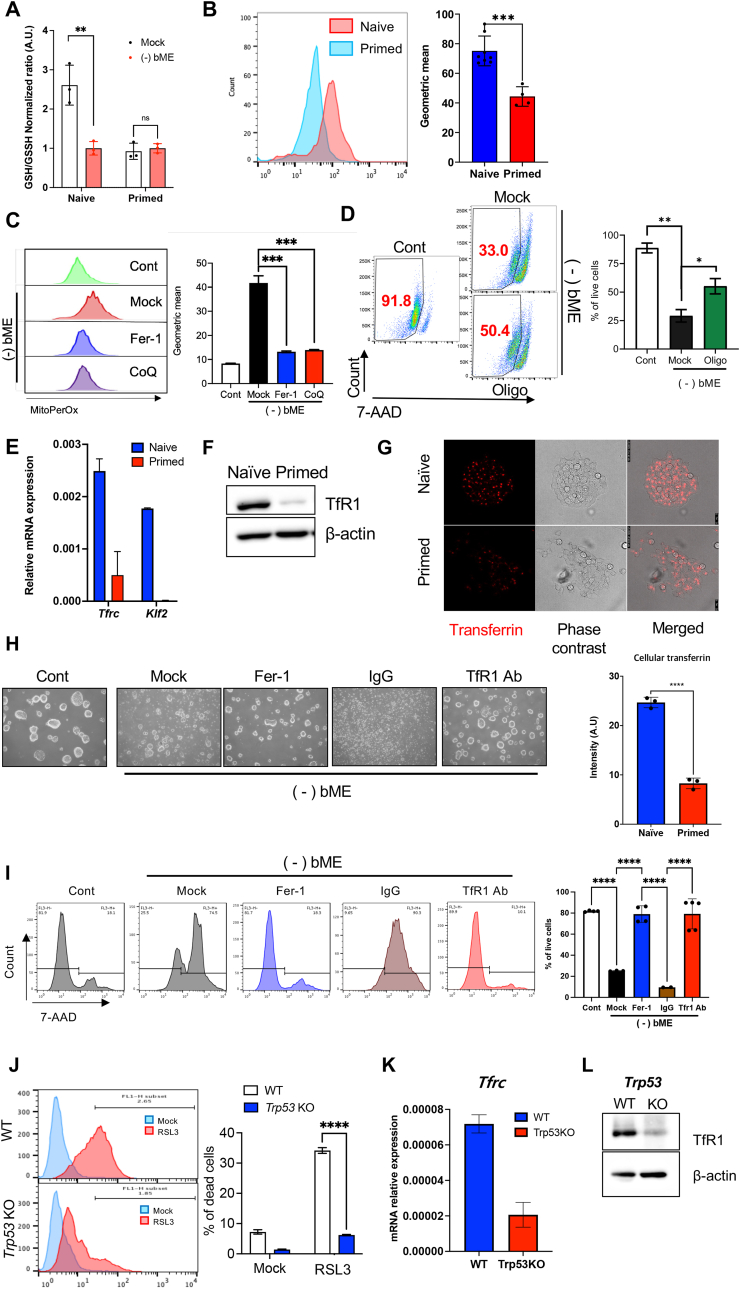
**Redox imbalance from oxidative phosphorylation and high TfR1 expression predispose naïve ESCs to ferroptosis (A)** Relative GSH/GSSG ratio in naïve and primed ESCs, cultured with and without bME for 24 h. Biological replicates: n = 3 per group. **(B)** Flow cytometric analysis of mitochondrial reactive oxygen species levels using mitoSOX (left) and quantification of the geometric mean fluorescence intensity (right) in naïve and primed ESCs in standard culture conditions. Biological replicates: n = 8 for naïve, and n = 4 for primed ESCs. **(C)** Flow cytometric analysis of mitochondrial lipid peroxidation in naïve ESCs at indicated culture conditions using mitoPerOX staining (left). bME-depleted cells were treated with Mock, Fer-1 (1 μM), or idebenone (CoQ, 2 μM) for 24 h. Quantification of geometric mean intensity is shown (right). Biological replicates: n = 3 per condition. **(D)** Viabilities of bME-depleted naïve ESC treated with Mock or oligomycin (Oligo, 1 μM) for 12 h (left). Quantification of the percentage of live cells is shown (right). Biological replicates: n = 3 per condition. **(E)***Tfrc* and *Klf2* expression in naïve and primed ESCs. Biological replicates: n = 3 per each group. **(F)** Immunoblot of TfR1 in naïve and primed mESCs. β-actin served as a loading control. **(G)** Representative images of fluorescence-labelled transferrin uptake in naïve (top row) and primed (bottom row) ESCs. Quantification of fluorescence intensity is shown in the bottom graph. Biological replicates: n = 3. **(H)** Representative microscopic images of control (Cont) and bME-depleted [(−)bME] naïve ESCs. In the bME-depleted group, cells were treated with Mock, Fer-1, control IgG, or anti-TfR1 antibody for 24 h. **(I)** Cell viability analysis of bME-depleted naïve ESCs treated with Mock, Fer-1, control IgG, or anti-TfR1 antibody for 24 h, assessed by 7-AAD staining (left). The percentage of live cells is shown (right). Biological replicates: n = 3 per condition. **(J)** Cell death of wild-type (WT) and *Trp53* knockout (KO) naïve ESCs treated with Mock or RSL-3 (0.5 μM), assessed by flow cytometry of 7-AAD staining (left). Quantification of the percentage of dead cells is shown (right). Biological replicates: n = 3 per condition. **(K)***Tfrc* expression in WT and *Trp53* KO naïve ESCs. Biological replicates: n = 3. **(L)** Immunoblot of TfR1 in WT and *Trp53* KO naïve ESCs. β-actin served as a loading control. Data are presented as mean ± s.d. **(A-E, G, I–K)**. Statistical significance was tested using two-tailed *t*-test **(A, B, G, J)** or one-way ANOVA followed by Tukey's post-hoc test **(C, D, I)**. ∗p < 0.05, ∗∗<0.01, ∗∗∗p < 0.001, ∗∗∗∗p < 0.0001; ns, not significant.

The marginal effect of statin treatment on primed ESCs suggests that higher MVA pathway activity and subsequent CoQ production are not sufficient to fully account for their distinct ferroptosis resistance (Fig. S2E). Among the various differences between naïve and primed pluripotent cells [3,4], the metabolic shift that occurs during embryonic development—where naïve ESCs exhibit high reliance on mitochondrial oxidative phosphorylation (OXPHOS) but primed ESCs predominantly utilize glycolysis [31]—stands out as a key feature that could determine susceptibility to ferroptosis. This metabolic distinction was clearly observed in the naïve and primed ESC pairs used in this study [6] and aligns with previous findings linking cellular metabolism to ferroptosis sensitivity [32]. Correspondingly, bME withdrawal resulted in significant decrease of the ratio of GSH to its oxidized form, GSSG, in naïve ESCs but not in primed ESCs (Fig. 4A). This observation suggests that certain intrinsic conditions of naïve pluripotency predispose such cells to oxidative stress. Furthermore, we found mitochondrial ROS production, as measured by MitoSox, to be markedly higher in naïve ESCs compared to primed ESCs (Fig. 4B). This result highlights that the more active oxidative phosphorylation (OXPHOS) in naïve ESCs compared to primed ESCs as seen in the previous study [6] contributes to a distinct redox imbalance, with mitochondria serving as a major source of intracellular ROS during ferroptosis [20,33,34]. Consistent with a previous demonstration [35], we showed the level of mitochondrial lipid peroxidation as determined by MitoPerOx [36] to correspond to ferroptotic events induced by bME depletion in naïve ESCs (Fig. 4C). Additionally, that cell death in naïve ESCs could be inhibited by perturbing mitochondrial electron transport chain (ETC) activity with oligomycin A, an ATP synthase inhibitor (Fig. 4D), supports the role of mitochondrial ETC activity in ferroptosis induction, as previously described [34]. These results suggest that the unique metabolic state of naïve ESCs, characterized by active mitochondrial OXPHOS, contributes to their heightened demand for a constant supply of antioxidants to prevent ferroptotic cell death.

**Fig.S3:**
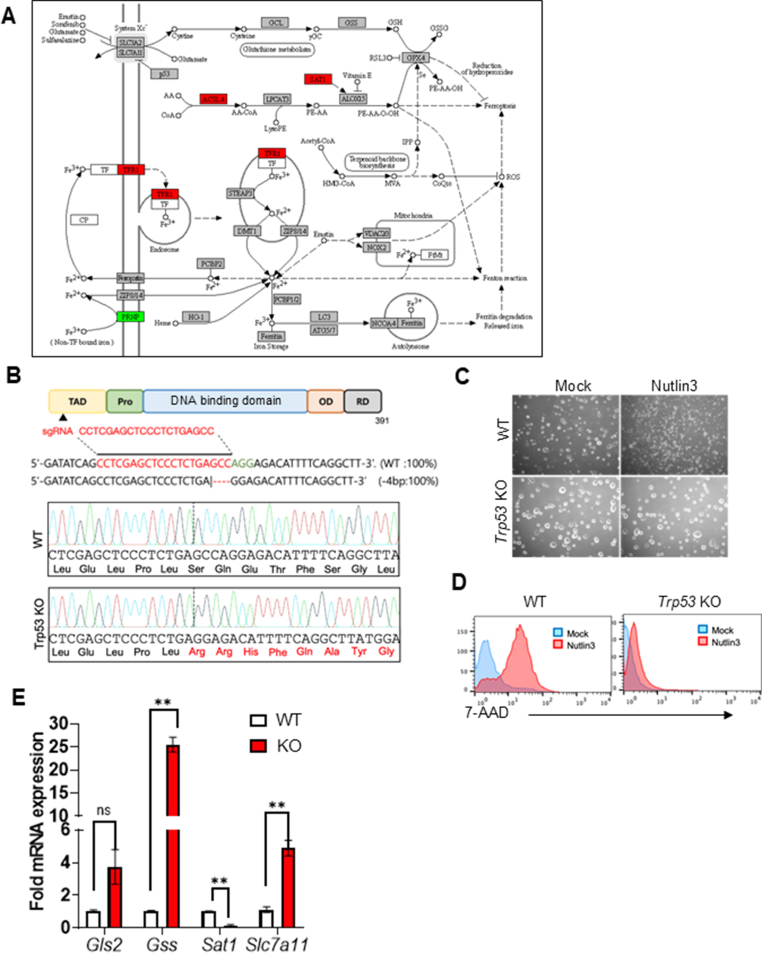


To identify the specific gene responsible for the heightened ferroptosis susceptibility in naïve ESCs, we analyzed differential gene expression between naïve and primed ESCs, focusing on genes included in the KEGG pathway *‘Ferroptosis’* (https://www.genome.jp/pathway/hsa04216) (Fig. S3A). Interestingly, we discovered that two genes essential for iron uptake, *Tf* (encoding transferrin) and *Tfrc* (encoding transferrin receptor, TfR1, also known as CD71), were significantly upregulated in naïve ESCs. The increased expression of TfR1 was validated through both qRT-PCR (Fig. 4E) and immunoblotting (Fig. 4F). Experimental assays also revealed high transferrin uptake in naïve ESCs compared to primed ESCs, which implies functionality of this TfR1 upregulation and possibly of iron ions in naïve ESCs (Fig. 4G). Notably, transferrin is one of the key components constantly supplied for pluripotency maintenance and the conversion to naïve ESCs [37]. In addition, iron supplementation promotes the formation of 8-cell embryos, morulae, and blastocysts, which provides indirect support for *Tf* and *Tfrc* induction in naïve ESCs [38]. Considering that TfR1 is also a key ferroptosis marker [39], we tested whether high expression of TfR1 and subsequent high iron ion uptake mediates ferroptosis in naïve ESCs. Notably, pre-treatment with blocking TfR1 antibodies entirely inhibited naïve ESC cell death post-bME deprivation, unlike the negative control IgG (Fig. 4H and I). Separately, we were able to reproduce the p53-dependent cell death observed in mESCs under bME withdrawal [15] in mouse induced pluripotent stem cells (iPSCs) when withdrawal was performed in the setting of *Trp53* knockout (*Trp53* KO or KO) (Fig. S3B) and treatment with Nutlin3, a stabilizer of p53 protein (Figs. S3C and S3D). In addition to *Trp53* KO cells exhibiting robust induction of NRF2-dependent antioxidant gene responses (Fig. S3E), consistent with previous findings [40], we observed marked repression of *TrR1* (Fig. 4K and L), which likely contributes to the high resistance of *Trp53* KO cells to RSL3-induced ferroptosis clearly evidenced in Fig. 4J.

### 3.5 The high GPX4 dependency of naïve ESCs is associated with oxidative stress

**Fig. 5:**
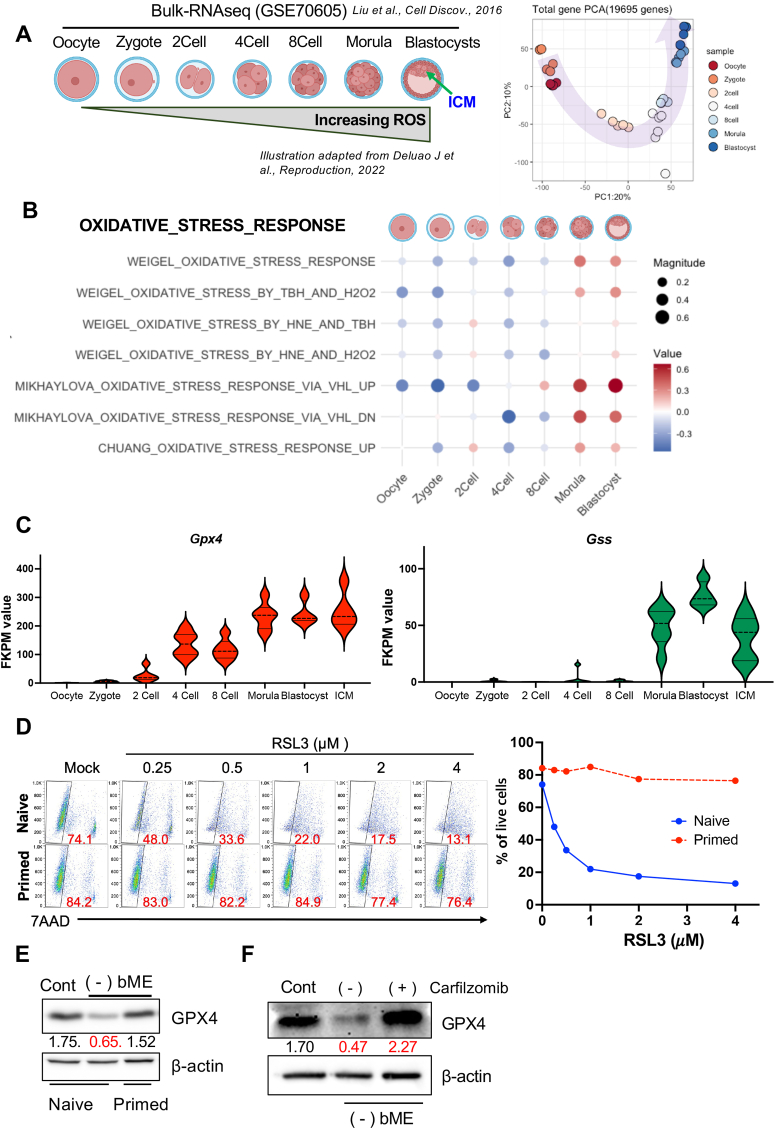
**The high GPX4 dependency of naïve ESCs is associated with oxidative stress (A)** Schematic representation of increasing ROS levels of ESCs from the oocyte to the inner cell mass (ICM) of blastocyst, adapted from Deluao J et al., 2022 (PMID: 36111646) (left). The ICM, the *in vivo* counterpart of naïve ESCs, is highlighted with an arrow. The PCA plot (right) illustrates the trajectory of transcriptional changes (red to blue) spanning embryonic developmental stages. Gene expression data were obtained from the GSE70605 dataset (Liu et al., 2016; PMID: 27462457). **(B)** Dot plot illustrating the enrichment of oxidative stress response gene sets from the Molecular Signatures Database (MSigDB) across developmental stages. Dot size represents the absolute enrichment score, indicating the magnitude of enrichment, while color indicates the direction of enrichment (blue for negative scores, red for positive scores). **(C)** Violin plots showing the expression levels (FPKM) of *Gpx4* (left) and *Gss* (right) across embryonic developmental stages. **(D)** Representative pseudocolor plots of 7-AAD stained naïve and primed ESCs treated with the indicated concentrations of RSL3 for 4 h (left). Red numbers indicate the percentage of 7-AAD negative live cells. Graphical representation of the percentage of live cells is shown (right). **(E)** Immunoblot analysis of GPX4 protein levels in naïve and primed ESCs cultured with (Cont) or without bME [(−) bME]. β-actin served as a loading control. The numbers represent the relative normalized densities. **(F)** Immunoblot analysis of GPX4 protein levels in bME-depleted naïve ESCs treated with carfilzomib for 24 h. β-actin served as a loading control. The numbers represent the relative normalized densities.

**Fig.S4:**
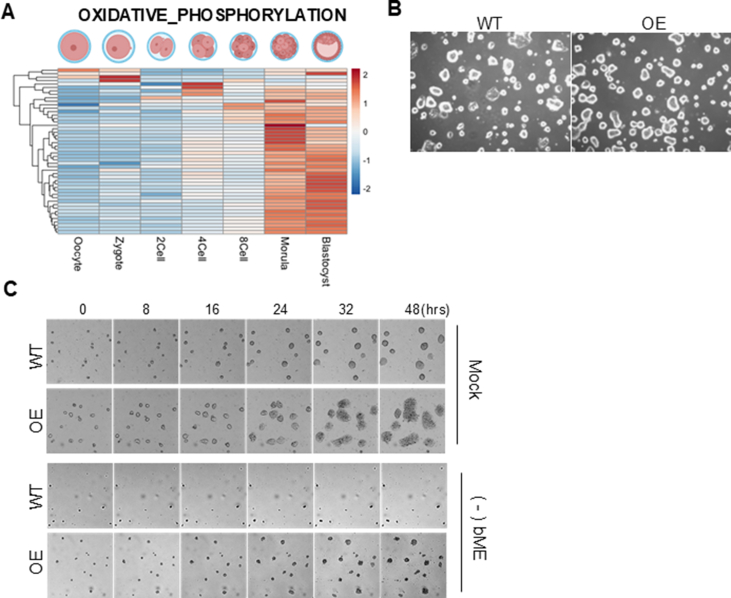


The increasing energy demands of the developing blastocyst, driven by rapid proliferation and differentiation, require high levels of ATP and correspondingly high biosynthesis, which leads to elevated ROS production [14] (Fig. 5A). Transcriptome analysis of pre-implantation embryo stages (GSE70605) [41] revealed enrichment of oxidative stress and oxidative phosphorylation gene sets at the morula and blastocyst stages (Fig. 5B and S4A). Correspondingly, transcript levels of *Gpx4* and glutathione synthase (*Gss*) gradually increased across these stages (Fig. 5C).

The high expression of *Gpx4* and *Gss* in the inner cell mass of blastocysts, whose properties are mirrored by naïve ESCs [42], highlights the dependency of naïve ESCs on GPX4 activity. Supporting this, naïve ESCs—but not primed ESCs—showed marked susceptibility to the GPX4 inhibitor RSL3 [43] or a TXNRD1 inhibitor [44]. Similar to cancer cells undergoing ferroptosis [45], GPX4 protein instability in naïve ESCs after bME depletion (Fig. 5E) was restored by treatment with carfilzomib, a proteasome inhibitor [21] (Fig. 5F).

### 3.6 Stable overexpression of GPX4 in naïve ESCs favors naïve pluripotency

**Fig. 6:**
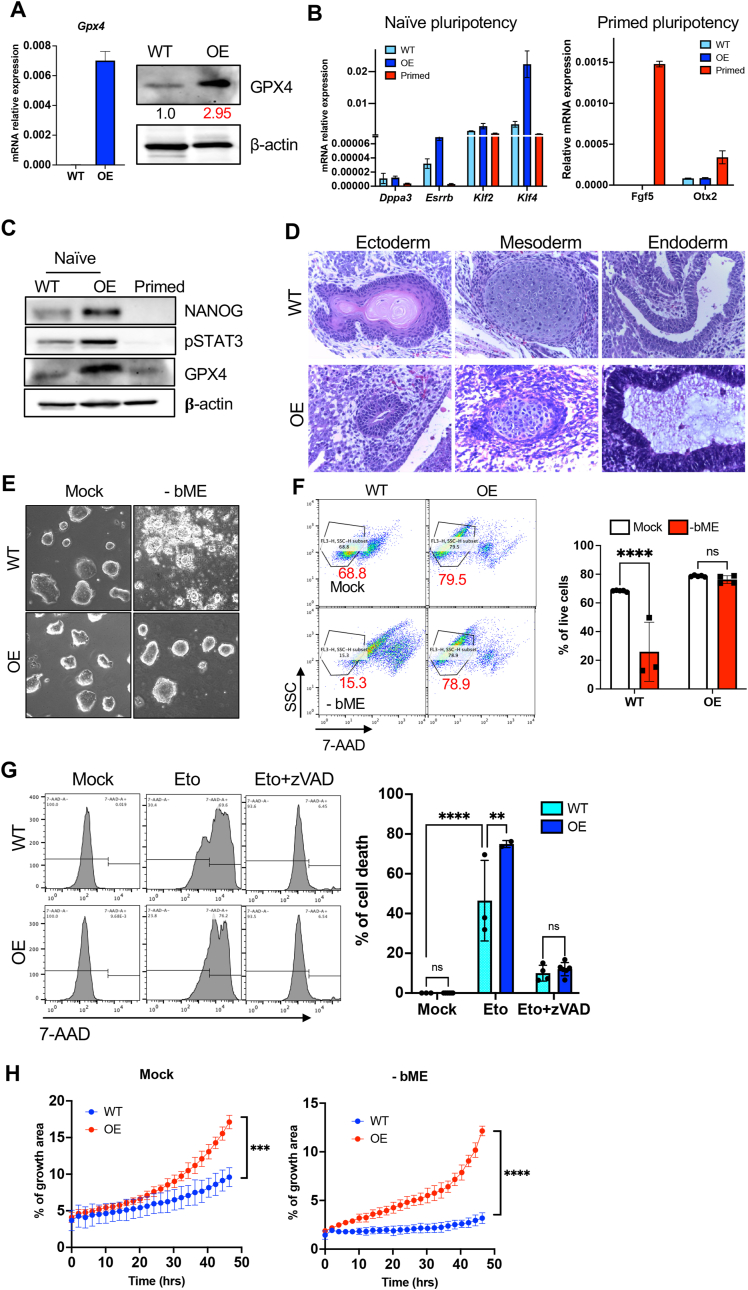
**Stable overexpression of GPX4 in naïve ESCs favors naïve pluripotency (A)** mRNA expression of *Gpx4* in wild-type (WT) and GPX4-overexpressing (OE) naïve ESC, assessed by qRT-PCR (left) (n = 3 biological replicates). Immunoblotting of GPX4 with β-actin as a loading control. The relative GPX4 protein level in OE cells (shown in red) was quantified by densitometry analysis using ImageJ. **(B)** Expression of naïve pluripotency-associated genes (*Dppa3, Esrrb, Klf2, Klf4*) (left) and primed pluripotency-associated genes (*Fgf5, Otx2*) (right) in WT and GPX4-OE naïve ESCs, compared with primed ESCs. Biological replicates: n = 3 per each group. **(C)** Immunoblot of NANOG, pSTAT3, and GPX4 in wild-type (WT) and GPX4-OE naïve ESCs compared with primed ESCs. β-actin served as a loading control. **(D)** Teratoma formation assay confirming pluripotency in WT and GPX4-OE naïve ESCs. The representative section images show all three germ layers. **(E)** Microscopic images of WT and GPX4-OE naïve ESCs cultured in the presence (Mock) or absence of bME [(−) bME]. **(F)** Cell viability of WT and GPX4-OE naïve ESCs in the absence of bME (-bME). Flow cytometric plots (left) show 7-AAD staining, with red numbers indicating the percentage of live cells. Quantification of the percentage of live cells is presented (right). Biological replicates: n = 3 per group. **(G)** Cell death analysis of WT and GPX4-OE naïve ESCs treated with etoposide (Eto, 1 μM) or etoposide plus zVAD (20 μM) (Eto + zVAD) for 24 h. Representative flow cytometric histograms (left) and quantification of 7-AAD positive dead cells (right) are shown. Biological replicates: n = 3 per group. **(H)** Quantification of growth area (%) of WT and GPX4-OE naïve ESCs cultured with (Mock, left) or without bME (-bME, right) over the indicated time. Biological replicates: n = 3 per group. Data are presented as mean ± s.d. **(A, B, F–H)**. Statistical significance was tested using two-tailed *t*-test **(F)**, one-way ANOVA followed by Tukey's post-hoc test **(G)**, or two-way ANOVA **(H)**. ∗∗<0.01, ∗∗∗p < 0.001, ∗∗∗∗p < 0.0001; ns, not significant.

Consistent with the high dependency of naïve ESCs on GPX4 activity (Fig. 5) and the embryonic lethality of homozygous *Gpx4* knockout [46], we were unable to establish *Gpx4* knockout ESCs despite multiple attempts (data not shown). Conversely, we successfully generated GPX4-overexpressing naïve ESCs (OE) with an approximately threefold increase in GPX4 protein expression compared to wild-type (WT) cells (Fig. 6A). These OE cells exhibited the typical dome-shaped morphology characteristic of naïve ESCs (Fig. S4B). Unexpectedly, these OE cells exhibited increased expression of naïve pluripotency markers such as *Esrrb* and *Klf4* (Fig. 6B), along with elevated NANOG protein levels and activation of STAT3 signaling [47], a key pathway for naïve pluripotency (Fig. 6C). A teratoma assay revealed OE cells to form a normal teratoma similar to WT ESCs, indicating minimal impact of stable GPX4 overexpression on development (Fig. 6D), consistent with observations in *Gpx4*-overexpressing mouse models [48].

In addition, stable *Gpx4* expression protected naïve ESCs from ferroptotic cell death under the bME-depleted condition (Fig. 6E and F), but did not rescue the apoptotic cell death induced by etoposide (Fig. 6G). Notably, OE cells displayed a growth advantage over WT cells under both bME-depleted and standard culture conditions (Fig. 6H and S4C), indicating that stable *Gpx4* expression promotes and sustains naïve pluripotency.

## 4 Discussion

Although ferroptosis has been extensively studied in cancer and degenerative diseases [18], its physiological role in embryo development remains uncharacterized, unlike apoptosis and other forms of programmed cell death [49]. Given the strong link between ferroptosis, metabolism, and redox state [18], the drastic changes in glucose metabolism and ROS production that occur in pre-implantation embryos [14] may necessitate protective mechanisms.

To investigate ferroptosis and corresponding protective mechanisms in pluripotent stem cells from pre- and post-implantation stages, we took as a model an isogenic pair of naïve and primed ESCs, which exhibit distinct cellular characteristics. A constant supply of bME has been widely accepted as essential for maintaining naïve pluripotency, and was previously suggested to protect against apoptosis induced by high oxidative stress [15]. However, our findings showed bME-depletion-induced cell death to be rescued by ferrostatin-1 (Fer-1) but not zVAD (Fig. 2), suggesting ferroptosis rather than apoptosis. Further evidence supporting ferroptosis as the operative mode included lipid peroxidation (Fig. 2C), absence of caspase-3 activity (Fig. 2E), and cell death inhibition by Fer-1 or CoQ analogs (Fig. 3G and H). This unique ferroptotic cell death in naïve ESCs results from constant ROS production and GSH oxidation (Fig. 4A and B) and is likely linked to their active OXPHOS metabolism [6], as it was rescued by ETC disruption (Fig. 4D). Distinct from primed ESCs, which rely on glycolysis [6,50], naïve ESCs require transferrin to supply iron ions to sustain ETC activity through TfR1 induction (Fig. 4G), further contributing to ferroptosis sensitivity (Fig. 4I). This metabolic shift from OXPHOS in naive ESCs to glycolysis and depletion of glycogen in primed ESCs [6] would activate the AMPK signaling pathway during the naive-to-primed transition [51] considering the direct inhibition of AMPK by glycogen [52]. The resulting changes in energy state and AMPK activity, which inhibit ferroptosis induction [53] may contribute to the selective ferroptosis sensitivity observed in naive ESCs, while potentially conferring protection against ferroptosis in primed ESCs.

The role of ROS in early embryo development, including in embryonic cleavage, compaction, blastulation, and blastocyst hatching, is well established [54]. As mitochondrial activity is ramped up during embryo development in accordance with energy demands, ROS levels progressively increase [55,56], necessitating robust antioxidant defenses that *in vivo* derive from enrichment of follicular and oviductal fluids in compounds such as vitamins A, C, and E, pyruvate, taurine, GSH, and cysteamine. This explains the need for antioxidant supplementation in *in vitro* culture media for embryos and naïve ESCs.

Consistent with this known role of ROS, transcriptome analysis revealed progressive increases in oxidative stress response and oxidative phosphorylation gene expression during pre-implantation development (Fig. 5A and B). Notably, *Gpx4* and *Gss* transcripts were induced at stages enriched for oxidative stress response gene sets (Fig. 5C), reflecting the dependency of naïve ESCs on GPX4 activity (Fig. 5D). These findings are corroborated by the enhanced naïve pluripotency and self-renewal observed upon stable GPX4 overexpression (Fig. 6).

The critical role of anti-ferroptosis mechanisms during embryo development is further exemplified by the fact that homozygous knockout of *Gpx4* or *Gss* in mice results in embryonic lethality at E7.5, prior to gastrulation in post-implantation embryos but not in pre-implantation embryos [46,57,58] unlike our observation. One notable discrepancy between *in vitro* culture conditions and the *in vivo* uterine environment is the presence of diverse anti-ferroptosis components in oviductal fluids, such as vitamins A [59] and E [60], taurine [61], and cysteamine [62] in the culture medium. The absence of these external anti-ferroptosis factors after implantation likely sensitizes *Gpx4* or *Gss* knockout embryos to ferroptosis at E7.5, which would account for the strong dependency of bME for culturing naïve ESCs *in vitro*.

In addition, supplementation with iron-chelating agents like EDTA [63] or sodium selenite [64] has been shown to improve *in vitro* embryo development in conditions where such anti-ferroptosis components are lacking, emphasizing the necessity of ferroptosis inhibition for early embryonic survival.

## 5 Conclusions

This study demonstrates that the survival of naïve ESCs depends on GPX4 activity, a dependence driven by their unique metabolic profile and high transferrin receptor expression for maintaining active OXPHOS. Consequently, bME supplementation is essential to prevent spontaneous ferroptosis of naïve ESCs during culture.

## CRediT authorship contribution statement

**Seokwoo Park:** Writing – original draft, Investigation, Formal analysis, Data curation. **Mihn Jeong Park:** Writing – original draft, Investigation, Formal analysis, Data curation. **Eun-Ji Kwon:** Formal analysis, Data curation. **Ji-Young Oh:** Validation, Data curation. **Yeon-Joon Chu:** Validation, Resources, Methodology. **Han Sun Kim:** Resources, Methodology. **Sunghyouk Park:** Supervision. **Tae Ha Kim:** Resources, Methodology. **Sung Won Kwon:** Supervision, Resources. **Yon Su Kim:** Supervision, Resources. **Hyuk-Jin Cha:** Writing – review & editing, Supervision, Project administration, Funding acquisition, Conceptualization.

## Funding

This work was supported by a grant from the National Research Foundation of Korea funded by Ministry of Science and ICT, Ministry of Health and Welfare (Grant number RS-2024-00432867 and RS-2023-00218543), a Research Grant from Seoul National University, and a grant No. 14-2024-0017 from the SNUBH Research Fund.

## Declaration of competing interest

The authors declare that they have no known competing financial interests or personal relationships that could have appeared to influence the work reported in this paper.

## Data Availability

Data will be made available on request.

## Abbreviations

GPX4: Glutathione peroxidase 4
ESCs: Embryonic stem cells
bME: β-mercaptoethanol
*Tfr1*: Transferrin receptor 1
mESCs: Mouse embryonic stem cells
hESCs: Human embryonic stem cells
OXPHOS: Oxidative phosphorylation
GSH: Glutathione
XCT: Imported cysteine through System XC
L-OOH: Toxic lipid peroxides
L-OH: Non-toxic lipid alcohols
FINs: Ferroptosis inducers
PUFA: Polyunsaturated fatty acid
LIFs: Leukemia inhibitor factors
bFGF: Basic fibroblast growth factors
Fer-1: Ferrostatin-1
ICM: Inner cell mass
TE: Trophectoderm
RSL3: (1S, 3R)-RSL3
MEF: Mouse embryonic fibroblasts
iOSKM: Inducible reprogramming factors
KO: Knockout
WT: Wild-type
EpiSCs: Epiblast stem cells
Srebps: Sterol-regulatory element binding proteins
CoQ: Coenzyme Q10
7-DHC: 7-dehydrocholesterol
PC: Phosphatidylcholine
USFAs: Unsaturated fatty acids
MS: Mass spectrometry
GSSH: Oxidized GSH
ROS: Reactive oxygen species
ETC: Electron transfer chain
NAO: 10-N-nonyl acridine orange
CL: Cardiolipin
CL-OOH: Cardiolipin hydroperoxide
Oligo: Oligomycin A

*Gss* Glutathione synthetase
