# Supplementary material for: The protective role of GPX4 in naïve ESCs is highlighted by induced ferroptosis resistance through GPX4 expression
Source: Redox Biol. 2025 Feb 10;81:103539. doi: 10.1016/j.redox.2025.103539 (PMC11908625; doi:10.1016/j.redox.2025.103539)
Supplement: Multimedia component 1 [file mmc1.docx]

**GPX4 expression protects from spontaneous ferroptosis from antioxidant deprivation in naïve ESCs**

Seokwoo Park^*^, Mihn Jeong Park^*^, Eun-Ji Kwon, Ji-Young Oh, Yeon-Joon Chu, Han Sun Kim, Sunghyouk Park, Tae Ha Kim, Sung Won Kwon, Yon Su Kim, Hyuk-Jin Cha

This PDF file includes:

Supplementary Materials and Methods

Supplementary Figure legends

Supplementary Figures

**Supplementary Materials and Methods**

**RNA extraction and RT-qPCR assay**

Total RNA was extracted using the easy-blue total RNA isolation kit (Intron Biotech, South Korea) according to the instruction. 0.5 to 1 µg of total RNA was converted to cDNA with the Prime Script RT reagent kit (Takara, Japan). TB-Green PCR reagent (Takara, Japan) was used to perform quantitative real-time PCR by LightCycler-480 II (Roche, Basel, Switzerland) per the manufacturer’s protocol. mRNA expression levels were normalized to the housekeeping genes Gapdh or Actb, which served as loading controls. Data were analyzed using the ΔΔCt method, and results are presented as relative expression levels

**Immunoblotting**

Cell lysates were extracted with RIPA buffer supplemented with 1% protease inhibitor cocktail and 0.1% sodium orthovanadate. After 1 hour incubation on ice, total protein was extracted after centrifugation. The concentration of total protein was quantified by BCA protein assay kit (Thermo Scientific). 10μg of total protein was separated on 10% SDS/PAGE. Separated protein in the gel was transferred to PVDF membrane. Membrane with protein was blocked with 10% skim milk in Tris-buffered saline containing 0.1% Tween-20 (TBS-T) for 1 hour and then washed three times by TBS-T. The membrane was incubated with primary antibody in TBS-T at 4°C overnight. Primary antibodies that were used in this study was described in a separate table. Incubated membrane was washed three times with TBS-T. The membrane was incubated at room temperature with HRP-conjugated secondary antibody in TBS-T for 1 hour, followed by wash with TBS-T three times. Immunoreactivity was detected by Chemi-Doc using WEST-Queen^TM^ kit (iNtRON Biotechnology, #16026).

**Flow cytometry**

The cells were detached using Accutase solution (BD Biosciences, #561527), followed by three washes with PBS. Subsequently, they were analyzed using either the FACS Calibur flow cytometry instruments from BD Bioscience. The flow cytometry data was analyzed using the CellQuest Pro or FlowJo software.

**Antibody information**

| **Antibody** | **Catalog** | **Company** |
| --- | --- | --- |
| β-actin | #47778 | Santa Cruz Biotechnology |
| pERK1/2 | #9101 | Cell signaling |
| pSTAT3 | #9145 | Cell signaling |
| GPX4 | ab125066 | Abcam |
| TFR1 | #269513 | Abcam |
| Nanog | #4903 | Cell signaling |

**RT-qPCR Primer sequence**

| **gene** | **Primer sequence (5’ to 3’)** |
| --- | --- |
| *18srRNA* | Fwd: CCA TCC AAT CGG TAG TAG CG  Rvs: GTA ACC CGT TGA ACC CCA TT |
| *Klf2* | Fwd: CAC ACA TAC TTG CAG CTA CAC CAA C  Rvs: CAA GTG GCA CTG AAA GGG TCT GTG |
| *Rex1* | Fwd: CTT CGA AAG CTT GGA GGA AGT GGA G  Rvs: GGA CAC TCC AGC ATC GAT AAG ACA C |
| *Tbx3* | Fwd: TCT CTA GCA TGG CTG CAG GCA TG  Rvs: CAG CCA TGT ATG TGT AGG GGT AAG |
| *Fgf4* | Fwd: GTT CTT CGT GGC CAT GAG CAG C  Rvs: CAG TCT AGG AAG GAA GTG GGT TAC |
| *Dppa3* | Fwd: CGT ACC TGT GGA GAA CAA GAG TG  Rvs: CAT TCT CAG AGG GAT CCC ATC TTT G |
| *Cer1* | Fwd: GTG GAA AGC GAT CAT GTC TCA TCG  Rvs: GCA AAG GTT GTT CTG GAC AAC GAC |
| *Fgf5* | Fwd: CAT CGG TTT CCA TCT GCA GAT CTA C  Rvs: GTT CTG TGG ATC GCG GAC GCA TAG |
| *Gls2* | Fwd: CCA TCG GCT ATT ACC TCA AGG AG  Rvs: TGC CTG ACT CAC AGG TAA CCT C |
| *Gss* | Fwd: CCA GGA AGT TGC TGT GGT GTA C  Rvs: GCT GTA TGG CAA TGT CTG GAC AC |
| *Sat1* | Fwd: GGC TAA ATT TAA GAT CCG TCC A  Rvs: CAT GTA TTC ATA TTT AGC CAG TTC CTT |
| *Slc7a11* | Fwd: CTG GGT GGA ACT GCT CGT AAT  Rvs: GTT CCA GGA TGT AGC GTC CA |
| *Hmgcr* | Fwd: GGC CCC ACA TTC ACT CTT GA  Rvs: ATC CAG CGA CTA TGA GCG TG |
| *Fdps* | Fwd: TTC TAC CTG CCT ATT GCG GC  Rvs: GGA AGA ACT CGC CCA TCT CC |
| *Fnta* | Fwd: TGA GGA GCA GCC CAA AAA CT  Rvs: ACT TCT CGC TCT AAC ACC GC |
| *Tfrc* | Fwd: TCT CCC GAG GGT TAT GTG GC  Rvs: AAG GTC TGC CTC AAC AAC GG |
| *Esrrb* | Fwd: GAT TCT CAT CTT GGG CAT CGT GTA C  Rvs: CTG ACT CAG CTC ATA GTC CTG CAG |
| *Nanog* | Fwd: GTG CAC TCA AGG ACA GGT TTC AG  Rvs: CTG CAA TGG ATG CTG GGA TAC TC |

**Supplementary Figure legends**

**Figure S1.** **(A)** Volcano plot of differentially expressed genes between the transcriptomes of isogenic pairs of naïve (J1 and OG2) and primed (PJ1 and PGO2) mESCs. Highlighted are key genes associated with the specific pluripotent states of naïve and primed ESCs. **(B)** Enrichment plots for the indicated gene sets. The gene sets were derived from differentially expressed genes specifically in naïve mESC compared with differentiated embryonic bodies representing primed state (reported by Hailesellasse Sene K. et al., 2007.). **(C)** Principal component analysis (PCA) of transcriptomes demonstrating distinct clustering of naïve mESCs (including J1, OG2, and meta-analyzed naïve ESCs) and their primed counterparts. Transcriptome datasets analyzed include GSE99491 (Bao S et al., 2018), GSE131555 (Kinoshita M et al., 2021), GSE105762 (Neagu A et al., 2020), and our own data. **(D)** Table summarizing cell lines utilized in this study (Fig. S1C), representing naïve- and primed-like states, along with annotations from previous reports. Transcriptome data were accessed using respective GSE numbers. **(E)** Immunoblot analysis of phosphorylated Stat3 (naïve specific) and Erk1/2 (primed-specific) in an isogenic pair of naïve and primed mESCs. 3T3 was used as a representative of differentiated control cell lines.

**Figure S2. (A)** Results of top enriched gene sets in primed ESCs, selected from Wikipathway gene set databases. Gene sets regarding cholesterol biosynthesis pathways were indicated in red. **(B)** Relative mRNA expressions of naive (left panel) and primed-specific (right panel) pluripotency markers in indicated cell lines. **(C)** Transcript levels of *Hmgcr*, *Fdps*, and *Fnta*, the MVA pathway genes, in indicated cell lines. **(D**) Transcript levels of *Hmgcr* and *Fdps* in naive and primed ESCs treated with vehicle or 1 μM atorvastatin for 12hours. **(E)** Effect of statin treatment on cell viability in naïve and primed ESCs. Representative microscopic images (left) and quantification of live cells by 7-AAD staining (right) are shown for cells treated with Mock or atorvastatin (1 μM). ns, not significant.

**Figure S3. (A)** Differentially expressed genes between the transcriptomes of naive (J1 and OG2) and primed (P-J1 and P-OG2) ESCs mapped on the ‘ferroptosis’ KEGG pathway (https://www.genome.jp/pathway/hsa04216). Genes upregulated in naïve ESCs are highlighted in red, while those downregulated are shown in green. **(B**) Graphical representation of the sgRNA-targeting site in the trans-activating domain (TAD) domain of wild-type (WT) mouse *Trp53* (upper panel). A 4-base pair deletion resulting in a frameshift was introduced in the knockout (*Trp53* KO) cell line, and this deletion was confirmed by Sanger sequencing (lower panel). TAD, trans-activating domain, Pro, proline-rich domain; OD, oligomerization domain; RD, C-terminal regulatory domain. **(C)** Representative phase contrast images of wild-type (WT) and *Trp53* knockout (KO) naïve iPSC cell lines treated with vehicle (Mock) or 10 μM nutlin3. **(D)** Flow cytometry of 7-AAD stained WT and *Trp53* KO cells treated with vehicle or nutlin3. **(E)** Relative mRNA expression levels of *Gls2*, *Gss*, *Sat1*, and *Slc7a11*, genes that are known to be modulated by p53, in WT or *Trp53* KO cells. **<0.01; ns, not significant.

**Figure S4.** **(A)** Heatmap illustrated the expression (FPKM) changes of genes involved in the Wikipathway Oxidative Phosphorylation pathway across various developmental stages. Rows represented individual genes, and columns corresponded to developmental stages. **(B)** Cell morphology of wild-type (WT) and GPX4-overexpressing (OE) naïve ESCs. **(C)** Microscopic images of wild-type (WT) and GPX4-OE naïve ESCs cultured in the presence (Mock, upper panels) or absence of bME [(-) bME, lower panels] over 48 hours.

**Movie S1.** Time lapse images of naïve ESCs **(A and B)** with **(A)** or without **(B)** bME supplement and primed ESCs **(C and D)** with **(C)** and without **(D)** bME supplement
